# The association of neighborhood racial mix and ED visit count in a cohort of patients on hemodialysis

**DOI:** 10.1186/s12882-019-1520-x

**Published:** 2019-09-02

**Authors:** Ladan Golestaneh, Atessa Farzami, Chikeluba Madu, Tanya Johns, Michal L. Melamed, Keith C. Norris

**Affiliations:** 1 Albert Einstein College of Medicine / Montefiore Medical Center, 3411 Wayne Ave, Suite 5H, Bronx, NY 10467 USA; 2 Albert Einstein College of Medicine, Bronx, NY USA; 3 David Geffen School of Medicine at University of California, Los Angeles, Los Angeles, CA USA

**Keywords:** Hemodialysis, Emergency department visits, Racial mix, Neighborhood, Disparities, End-stage kidney disease

## Abstract

**Background:**

Neighborhood racial mix is associated with dialysis facility performance metrics and mortality outcomes in patients on hemodialysis. We explored the association of neighborhood racial mix with emergency department (ED) visits in patients receiving hemodialysis.

**Methods:**

Using Looking Glass (Montefiore’s clinical database) we identified a cohort of patients on hemodialysis with an index ED visit at any of 4 Montefiore Hospital locations, between January 2013 and December 2017 and followed it for number of ED visits through December of 2017 or dropout due to death. The racial mix data for the Bronx block group of each subject’s residence was derived from the Census Bureau. We then used negative binomial regression to test the association of quintile of percent of Black residents per residential block group with ED visits in unadjusted and adjusted models. To adjust further for quality offered by local dialysis facilities, with the facility zip code as the locus, we used data from the “Dialysis Compare” website.

**Results:**

Three thousand nine-hundred and eighteen subjects were identified and the median number of ED visits was 3 (interquartile range (IQR) 1–7) during the study period. Subjects living in the highest quintile of percent Black residents were older, more commonly female and had lower poverty rates and higher rates of high school diplomas. Unadjusted models showed a significant association between the highest quintiles of Black neighborhood residence and count of ED visits. Fully adjusted, stratified models revealed that among males, and Hispanic and White subjects, living in neighborhoods with the highest quintiles of Black residents was associated with significantly more ED visits (p-trend =0.001, 0.02, 0.01 respectively). No association was found between dialysis facility locations’ quintile of Black residents and quality metrics.

**Conclusions:**

Living in a neighborhood with a higher percentage of Black residents is associated with a higher number of ED visits in males and non-Black patients on hemodialysis.

**Electronic supplementary material:**

The online version of this article (10.1186/s12882-019-1520-x) contains supplementary material, which is available to authorized users.

## Background

The association of health outcomes with neighborhood residence has long been described as an important manifestation of racial segregation in the US [1–5]. Disparities in access to optimal healthcare services are cited as reasons for poor health outcomes, while other reasons relate to lack of neighborhood resources to support healthy habits and diets, exposure to crime and environmental toxins, and limited access to high quality outpatient care coordination [1, 5]. Whether these disparities are related to socioeconomic disadvantages in predominantly Black or Hispanic neighborhoods, institutional/structural racism inherent in political and social systems, and/or local healthcare practices is a point of controversy [5–9]. It is difficult to separate socioeconomic disadvantage from the racial/ethnic makeup of most metropolitan neighborhoods. Therefore, socioeconomic and race/ethnicity factors as they relate to health outcomes are frequently clumped together [1, 4].

Neighborhood characteristics determine health outcomes in the end-stage kidney disease (ESKD) population as well [8, 10–15]. Black and White patients that receive care in dialysis facilities located in neighborhoods with a higher percentage of Black residents are less likely to meet performance benchmarks as defined by the Centers for Medicare Services (CMS) [16]. Furthermore, there are higher rates of hospitalizations in patients from dialysis facilities located in areas with a higher percentage of Black residents [16]. Black patients have lower rates of access to pre-ESKD care by a nephrologist, are less likely to have a fistula as the first dialysis access, and have lower rates of referral to transplant centers, which explains some of these outcomes [16, 17]. This is of particular interest because despite these disadvantages, Black patients on hemodialysis have longer adjusted survival time as compared to Whites [16, 18–20]. CMS had proposed adjusting for case mix based on neighborhood characteristics when calculating benchmarks for standardized readmission ratios as part of the quality incentive program. However, when adjusted for a composite neighborhood score (ADI: area deprivation index) the readmission rates studied by CMS were not altered to a degree that they felt warranted altering the existing case mix formula and as a result, neighborhood characteristics were left out of benchmark determinations [21].

In this study, we explore the association of neighborhood racial/ethnic make-up, with subjects’ residence as the locus for the census block group, with emergency department (ED) visits in a cohort of patients on hemodialysis that live in the Bronx. We hypothesize that the neighborhood racial/ethnic makeup alone, independent of socio-economic status, is not associated with ED visits. To reduce residual confounding we did a separate analysis, wherein we evaluated the association of neighborhood racial/ethnic make-up with dialysis facility characteristics.

## Methods

The study was approved by the Albert Einstein Institutional Review Board. We adhered to the Declaration of Helsinki in our methods.

### Sources of data

**Fig. 1:**
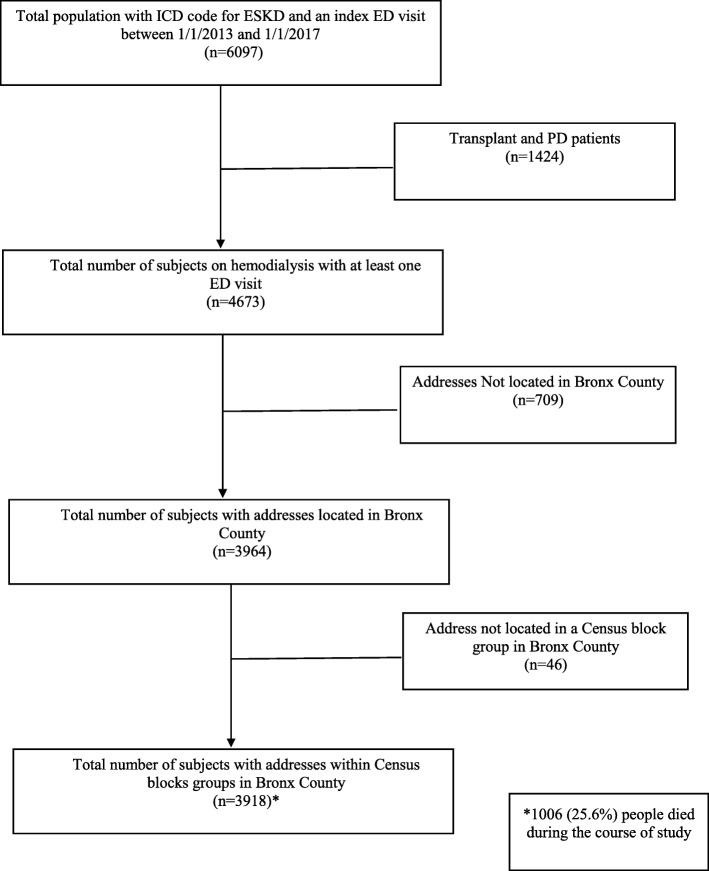
Study Flow Chart

Using Looking Glass™, we identified a cohort of patients on hemodialysis who had an index ED visit between 2013 and 2017 (1/1/2013–1/1/2017), at any of four Montefiore Hospitals in the Bronx, NY. Looking Glass™ Clinical Analytics (Streamline Health, Atlanta, Georgia) is a software application developed at Montefiore to help build a clinical database. It integrates clinical and administrative datasets to identify the specific patient cohorts and allows for retrospective measurement of our outcome [22, 23]. We included only adult patients (age > 21) who were receiving maintenance hemodialysis, with a mix of incident and prevalent hemodialysis patients. The cohort was identified using ICD 9 and 10 codes for ESKD, transplant recipients were excluded using ICD 9 and 10 codes (including those returning to dialysis) while patients on peritoneal dialysis (PD) were excluded using PD billing codes (Fig. 1). For this analysis we excluded all ED visits that led to hospital admissions, choosing instead to focus on ED visits that did not lead to an admission [22]. We linked subject level variables and American Community Survey (ACS) data (percent Black, percent living under the poverty line, percent graduated from high school) by using subjects’ home addresses as the locus of the block group. We used the United States Census Bureau, 2012–2016 ACS 5-Year Estimates to obtain neighborhood characteristics [24] (Fig. 1). There were 954 block groups in the Bronx. Compared to those that had a census linked block group, those subjects that did not (*n* = 683) were slightly younger (mean age 58.0 years (standard deviation (SD) 15.6)), had the following racial/ethnic breakdown: Whites 83 (11.7%), Blacks 311 (43.9%), Hispanics 250 (35.3%) and “other” 65 (9.2%), and were more commonly male (428 (60.4%)). Our sample size had missing data points for the body mass index variable (526 missing) representing < 15% of the total data points, with the missingness completely at random, as tested by Little’s MCAR test. Therefore a multiple imputation strategy was not used [25].

### Dialysis facility data

We did a separate analysis to evaluate differences in quality of dialysis delivery between dialysis facilities located in the Bronx. We used the “Dialysis Compare” database provided by CMS to identify the location, ownership and performance on CMS selected quality measures. We linked ACS indicators to the census tract for each dialysis facility address using the “geocoding” application. We then analyzed the association of dialysis facility block group attributed percent of Black residents, percent living under the poverty line and percent that graduated high school, with CMS reported hospitalization rates for 2016 for that facility, and five star ratings reported by each facility to CMS for the year 2016.

### Primary exposure

We categorized our exposure variable, percent Black residents in a block group, into quintiles for the final analysis as follows: 1 = 1.0–19.1% (reference group); 2 = 19.2–29.3%; 3 = 29.5–45.8%, 4 = 46.4–68.9%; 5 = 69.3–100%.

### Primary outcome

We obtained longitudinal data on ED visits until the end of the study period or death event. We excluded all ED visits that led to hospital admissions, choosing instead to focus on ED visits that did not lead to an admission. Previous studies have shown a strong association between previous ED visits and repeat ED utilization, demonstrating a pattern of behavior in response to lack of outpatient engagement [26–28].

### Covariates

Patient-level covariates included: 1- demographic (age, sex, race, ethnicity, socioeconomic status, primary language (English vs not-English), marital status, type of residence (skilled nursing facility (SNF) vs non- SNF) and insurance status (Commercial, Medicaid, Medicare), and 2- clinical/anthropomorphic (Charlson score, presence of permanent catheter (permcath)) for dialysis, dialysis relevant laboratory values that are validated prognostic markers included minimum albumin within 90 days around index ED visit, lowest and highest serum phosphorus levels within 90 days around index ED visit, lowest and highest hemoglobin (Hgb) levels within 90 days around index ED visit, minimum body mass index (BMI) within 90 days around index ED visit, history of diabetes mellitus (defined as HgbA1c > 6.0% or an ICD diagnosis of diabetes mellitus within 1 year prior to index ED visit), and history of heart failure (defined as any ICD diagnosis associated with heart failure within 1 year prior to index ED visit). The Charlson score was calculated by Looking Glass based on ICD codes [29] (Additional file 1). Individual socio-economic status (SES), is represented by Looking Glass after it is derived from Census Bureau Tract data and the results represent the standard deviation above or below New York State’s mean income [3].

### Statistical analysis

We used STATA version 15.0 for all analyses. The association of demographic and clinical variables and quintile of percent of Black residents attributed to block group was tested using one-way ANOVA and Kruskal Wallis tests for continuous variables and Chi-Square or Fisher’s exact tests for categorical variables. We used negative binomial regression to test the association of each variable with the outcome: ED visit count. We adjusted this analysis to duration of study follow-up, using date of death as the dropout date. We then built a model using negative binomial regression to test the association of quintile of percent of Black residents with ED visits in our cohort, while adjusting for 1) sociodemographic variables that were significant in bivariate analysis only, and 2) sociodemographic and clinical variables that were significant in bivariate analyses.

We tested the following for interactions with respect to the outcome of ED visits by placing a multiplicative term in the model: quintile of percent Black residents and subjects’ SES, quintile of percent Black residents and sex, quintile of percent Black residents and race category of subjects, quintile of percent Black residents and insurance status of subjects. Because of significant interactions found between at least one quintile (#3) of percent Black residents and sex (*p* = 0.01) as well as quintile (#3) of percent Black residents and self-identified race (0.02) we stratified all multivariable models based on sex and race.

## Results

### Study population

Six thousand and ninety-seven subjects with a diagnosis of ESKD had made at least one ED visit to any of 4 Montefiore affiliated health facilities in the Bronx. Four thousand six hundred and seventy-three of these were on hemodialysis and 3918 who had complete data on Census Bureau and clinical attributes were identified (Fig. 1). The mean age of the population was 62.2 years (+/− 14.8), 1853 (46.7%) were Black, 1539 (38.8%) were Hispanic, and 260 (6.6%) were White (Table 1). Two-thousand, two hundred and twenty-six subjects (56.2%) were male, and 812 (20.5%) did not speak English as their primary language. The median number of ED visits made by each patient after the index ED visit for the duration of the study period (median: 2.69 years (IQR: 1.36–3.56)) was 3 (interquarile range (IQR) 1–7). The median Charlson score was 5 (IQR 2–7).

**Table 1:** Baseline characteristics of the study population

	Quintiles of Black Residents by Block Group Residence (*N* = 3918)					
	Q1 (1.0–19.1%^e^) *n* = 743	Q2 (19.2–29.3%^e^) *n* = 803	Q3 (29.5–45.8%^e^) *n* = 849	Q4 (46.4–68.9%^e^) *n* = 736	Q5 (69.3–100%^e^) *n* = 787	*P*-Value
Demographics						
Age, years (mean (SD))	61.5(14.4)	61.7 ± 14.6	61.7 ± 14.6	60.9 ± 14.9	64.9 ± 15.0	< 0.001
Female (n(%))	294 (39.6)	349 (43.5)	372 (43.8)	326 (44.3)	379 (48.2)	0.02
Race/Ethnicity (n(%))						
Non-Hispanic White	86 (11.6)	45 (5.6)	40 (4.7)	38 (5.2)	38 (4.8)	< 0.001
Non-Hispanic Black	200 (26.9)	289 (36.0)	389 (45.8)	402 (54.6)	563 (71.5)	
Hispanic	362 (48.7)	401 (49.9)	368 (43.3)	253 (34.4)	138(17.5)	
Other	95 (12.8)	68 (8.5)	52 (6.1)	43 (5.8)	48(6.1)	
English- speaker (n(%))	518 (69.7)	576 (71.7)	665 (78.3)	618 (84.0)	736 (93.5)	< 0.001
Socioeconomic variables						
Percent Living below the Poverty line (median % [IQR])	29.6 [15.1–39.4]	34.7 [22.4–41.5]	35.2 [20.5–48.4]	29.8 [23.8–40.9]	14.3 [7.4–20.5]	< 0.001
Mean Percent Graduated High School in the Area (percent (SD)	69.1 ± 14.7	63.0 ± 13.5	65.1 ± 12.4	71.0 ± 11.8	80.4 ± 7.8	< 0.001
Married(n(%))	286 (38.5)	294 (36.6)	257(30.3)	228(31.0)	273(34.7)	0.002
State ADI rank (median (IQR))	4 [2–6]	3 [2–6]	2 [2–6]	5 [2–6]	4 [2–7]	< 0.001
SES (median (IQR))	−3.39 [− 6.20-(− 1.04)]	−3.87 [− 6.40-(− 2.32)]	−4.06 [− 6.70-(− 2.38)]	−5.06 [− 7.02-(− 1.54)]	−1.98 [− 1.42-(− 0.98)]	< 0.001
Insurance (n(%))						
Commercial	85 (12.3)	101 (13.6)	97 (12.2)	93 (13.7)	103 (13.8)	0.05
Medicaid	289 (41.8)	292 (39.3)	303 (38.1)	273 (40.3)	246 (33.0)	
Medicare	317 (45.9)	349 (47.0)	395 (49.7)	312 (46.0)	396 (53.2)	
Clinical Variables						
Living in SNF (n(%))	62 (9.0)	89 (11.9)	77 (9.7)	55 (8.1)	105 (14.0)	0.001
Diabetes Mellitus (n(%))	444 (59.8)	452 (56.3)	470 (55.4)	434 (59.0)	472 (60.0)	0.20
Heart Failure (n(%))	243 (32.7)	262 (32.6)	263 (31.0)	237 (32.2)	268 (34.0)	0.77
Charlson Score (median (IQR))	4 [2–7]	5 [2–7]	5 [2–7]	5 [2–7]	5 [3–8]	0.01
Permanent Catheter (n(%))	169 (22.7)	197 (24.5)	195 (23.0)	129(17.5)	165(21.0)	0.01
^a^BMI, Kg/m^2^ (mean (SD))	26.9 ± 6.95	27.6 ± 9.4	27.0 ± 9.5	27.2 ± 7.5	26.6 ± 6.9	< 0.001
^b^Serum Albumin, mg/dL (mean (SD))	3.77 ± 0.58	3.72 ± 0.61	3.73 ± 0.61	3.76 ± 0.63	3.74 ± 0.57	0.52
^c^Min phosphorus, mg/dL (mean (SD))	3.6 ± 1.39	3.6 ± 1.46	3.56 ± 1.48	3.62 ± 1.38	3.55 ± 1.48	0.25
^c^Max phosphorus, mg/dL (mean (SD))	6.12 ± 2.41	5.97 ± 2.20	5.97 ± 2.21	5.90 ± 2.30	5.90 ± 2.24	0.10
^d^Max Hemoglobin, mg/dL (mean (SD))	11.5 ± 1.78	11.5 ± 1.83	11.5 ± 1.86	11.6 ± 1.78	11.4 ± 1.72	0.24
^d^Min Hemoglobin, mg/dL (mean (SD))	9.16 ± 2.30	9.14 ± 2.27	9.02 ± 2.23	9.33 ± 2.27	8.88 ± 2.11	0.19
ED visits						
Number of visits per patient over the duration of the study (median (IQR))	3 (1–6)	3 (1–7)	3 (1–6)	3(1–7)	4 (1–8)	< 0.001

Values for categorical variables are given as count (proportion); values for continuous variables are given as mean ± standard deviation or median [interquartile range] if skewed

*Abbreviations*: *ADI* Area deprivation index, *SES* Socioeconomic status, *BMI* Body mass index, *SNF* Skilled nursing facility, *ED* Emergency department

Complete data available except for the following variables: ^a^BMI (*n* = 3369), ^b^Phosphorus (*n* = 3336), ^c^Albumin (*n* = 3606), ^d^Hemoglobin (*n* = 3742)

^e^Median

Of the block groups examined (in which at least one subject lived) the percent of Black residents ranged from 2.6 to 90.3% of the block group population, with the median percent Black residents at 36.1% (IQR: 23.3–60.9%). Subjects living in the highest quintile of percent Black residents were older and more commonly female. The highest percent Black block groups had fewer Hispanics, and fewer non-English speaking patients on hemodialysis as compared to subjects living in the lower percent Black population quintiles (Table 1). Interestingly, fewer subjects living in the highest percentage of Black neighborhoods in the Bronx were living under the poverty line, and in general this population had a higher percentage of high school graduates and had higher SES (Table 1).

### Associations between baseline characteristics and ED visits

Bivariate analysis showed that the following variables had a significant association with high incidence rate ratios (IRR) of avoidable ED visits: quintiles 4 and 5 of percent Black residents, non-English as primary language, Medicaid insurance, a history of heart failure, a higher Charlson score, and higher maximum phosphorus levels (Table 2). In contrast, being older, living in a SNF, having higher minimum phosphorus, having higher BMI, having higher minimum albumin and having a higher minimum hemoglobin were all significantly protective against an increased IRR for ED visits.

**Table 2:** Association of socio-demographic and clinical variables with Emergency Department (ED) visits

Variable	IRR for number of future ED visits	95% confidence Interval
Proportion of Black residents in community (*n* = 3918)		
Q1	1.00	
Q2	1.08	0.96–1.22
Q3	1.02	0.91–1.14
Q4	1.15	1.02–1.30
Q5	1.15	1.03–1.30
Graduated High school (*n* = 3918)		
Q1	1.00	
Q2	1.03	0.92–1.15
Q3	1.07	0.96–1.20
Q4	1.10	0.98–1.24
Q5	0.98	0.87–1.10
% living under poverty line (*n* = 3918)		
Q1	1.0	
Q2	1.06	0.94–1.19
Q3	1.15	1.02–1.29
Q4	1.15	1.02–1.29
Q5	0.96	0.86–1.08
Age (*n* = 3964)		
Q1 (reference)	1	
Q2	0.90	0.81–1.01
Q3	0.90	0.81–1.01
Q4	0.85	0.76–0.96
Q5	0.88	0.79–0.99
Gender (*n* = 3964)		
Male	1.0	
Female	1.07	0.99–1.15
Race (*n* = 3964)		
Non-Hispanic White	1.0	
Non-Hispanic Black	1.16	0.99–1.36
Hispanic	1.16	0.99–1.35
Other	0.96	0.78–1.17
Language (*n* = 3964)		
English	1.0	
Non-English	1.10	1.00–1.20
Insurance (*n* = 3695)		
Commercial	1.0	
Medicare	1.06	0.95–1.18
Medicaid	1.16	1.03–1.29
Married (*n* = 3964)		
Not married	1.0	
Married	0.93	0.86–1.01
Lived in SNF (*n* = 3715)		
No	1.0	
Yes	0.77	0.67–0.88
PC use at index ED visit (*n* = 3964)		
No	1	
Yes	1.06	0.97–1.16
History of DM (3964)		
No	1.0	
Yes	0.98	0.91–1.05
History of Heart Failure (*n* = 3964)		
No	1.0	
Yes	1.23	1.14–1.33
Charlson Score	1.02	1.00–1.03
BMI (*n* = 3997)	0.99	0.98–0.99
Min phosphorus (*n* = 3969) (for every 1 mg/dL increase in value)	0.94	0.92–0.97
Max phosphorus (*n* = 3969) (for every 1 mg/dL) increase in value)	1.04	1.02–1.06
Min albumin (*n* = 3606) (for every 1 g/dL increase in value)	0.87	0.81–0.94
Max Hemoglobin (*n* = 3784) (for every 1 g/dL increase in value)	1.01	0.99–1.03
Min Hemoglobin (*n* = 3784) (for every 1 g/dL increase in value)	0.93	0.92–0.94

*DM* Diabetes mellitus, *SNF* Skilled nursing facility, *IRR* Incident rate ratio, *BMI* Body mass index, *PC* Permacath

### Associations between neighborhood percent black residents and ED visits

**Fig. 2:**
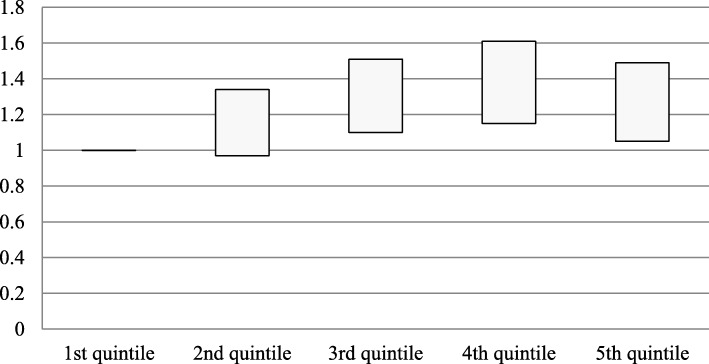
IRR of Avoidable ED Visit Count and Quintile of Percent Black in Neighborhood of Residence in Male Patients on Hemodialysis

The association between quintile of percent Blacks in the population and repeat ED visits was significant only in men, and addition of socio-demographic and clinical variables to the model strengthened this association (Table 3; Fig. 2). Furthermore, for White patients on hemodialysis, the association with repeat ED visits was robust especially in the quintile 4 and 5 (though not statistically significant in quintile 5) after adjusting for socio-demographic and clinical variables (Table 4). For Hispanic subjects on hemodialysis, the association of increasing quintile (quintiles 4 and 5) of percent Black in the block group with ED visits was significant and strengthened with the addition of socio-demographic and clinical variables, the latter of which suggests a possible mediation effect by these variables.

**Table 3:** Incident Rate Ratio (IRR) for Emergency Department (ED) visits by quintile of percent black population stratified by sex

Sex	% Black in the Block Area	Model 1: Unadjusted IRR	Model 2: IRR adjusted for socio-demographic variables^a^	Model 3: IRR adjusted for socio-demographic and clinical variables^b^
Male	Q1	1.0 (Reference)	1.0 (Reference)	1.0 (Reference)
	Q2	1.07 (0.92–1.25)	1.09 (0.93–1.27)	1.15 (0.97–1.34)
	Q3	1.16 (1.00–1.35)	1.19 (1.02–1.39)	1.29 (1.10–1.51)
	Q4	1.25 (1.07–1.46)	1.28 (1.09–1.51)	1.36 (1.15–1.61)
	Q5	1.20 (1.03–1.40)	1.21 (1.03–1.42)	1.25 (1.05–1.49)
	p-trend	0.004	0.003	0.001
Female	Q1	1.0 (Reference)	1.0 (Reference)	1.0 (Reference)
	Q2	1.07 (0.89–1.28)	1.07 (0.89–1.29)	1.12 (0.92–1.36)
	Q3	0.85 (0.71–1.02)	0.86 (0.72–1.03)	0.89 (0.72–1.05)
	Q4	1.03 (0.86–1.24)	1.04 (0.86–1.25)	1.15 (0.93–1.39)
	Q5	1.08 (0.90–1.29)	1.18 (0.98–1.42)	1.21 (0.97–1.46)
	p-trend	0.48	0.20	0.10

^a^ Adjusted for age, race/ ethnicity, primary language, neighborhood percent poverty, neighborhood percent graduated from high school, percent married, SES, and insurance

^b^ Adjusted for all of above + presence of permcath, residence at skilled nursing facility, Charlson score, minimum Hemoglobin and body mass index within 90 days of index ED visit

**Table 4:** Incident Rate Ratio (IRR) for Emergency Department (ED) visits by quintile of percent black population stratified by race/ ethnicity

Race/Ethnicity	% Black in the Block Area	Model 1: Unadjusted IRR	Model 2: IRR adjusted for socio-demographic variables^a^	Model 3: IRR adjusted for socio-demographic and clinical variables^b^
Non-Hispanic White (247)	Q1	1.0 (Reference)	1.0 (Reference)	1.0 (Reference)
	Q2	1.01 (0.64–1.60)	0.92 (0.59–1.51)	0.94 (0.55–1.60)
	Q3	1.29 (0.81–2.04)	1.16 (0.69–1.94)	1.18 (0.68–2.03)
	Q4	1.75 (1.12–2.73)	2.09 (1.21–3.60)	2.29 (1.27–4.12)
	Q5	1.16 (0.72–1.83)	1.16 (0.72–1.88)	1.31 (0.78–2.20)
	p-trend	0.07	0.09	0.02
Non-Hispanic Black (1842)	Q1	1.0 (Reference)	1.0 (Reference)	1.0 (Reference)
	Q2	0.97 (0.79–1.20)	1.00 (0.81–1.23)	1.10 (0.87–1.38)
	Q3	1.00 (0.82–1.21)	1.00 (0.82–1.22)	1.10 (0.88–1.36)
	Q4	1.00 (0.82–1.22)	1.00 (0.82–1.22)	1.10 (0.89–1.37)
	Q5	1.04 (0.86–1.25)	1.14 (0.93–1.39)	1.18 (0.95–1.47)
	p-trend	0.49	0.20	0.15
Hispanic (1522)	Q1	1.0 (Reference)	1.0 (Reference)	1.0 (Reference)
	Q2	1.11 (0.94–1.30)	1.17 (0.99–1.39)	1.22 (1.03–1.45)
	Q3	0.93 (0.78–1.10)	0.99 (0.83–1.17)	1.03 (0.86–1.23)
	Q4	1.19 (0.99–1.43)	1.24 (1.03–1.50)	1.36 (1.11–1.69)
	Q5	1.26 (1.00–1.58)	1.26 (0.99–1.59)	1.37 (1.07–1.76)
	p-trend	0.07	0.06	0.01
Other (306)	Q1	1.0 (Reference)	1.0 (Reference)	1.0 (Reference)
	Q2	1.12 (0.79–1.59)	1.15 (0.79–1.67)	1.13 (0.77–1.64)
	Q3	0.97 (0.66–1.44)	1.03 (0.70–1.51)	0.96 (0.63–1.44)
	Q4	1.21 (0.82–1.80)	1.29 (0.85–1.94)	1.26 (0.82–1.95)
	Q5	1.29 (0.89–1.89)	1.32 (0.89–1.97)	1.18 (0.77–1.80)
	p-trend	0.17	0.16	0.52

^a^ Adjusted for age, sex, primary language, neighborhood percent poverty, neighborhood percent graduated from high school, percent married, SES, and insurance

^b^ Adjusted for all of above + presence of permcath, residence at skilled nursing facility, Charlson score, minimum Hemoglobin and body mass index within 90 days of index ED visit

### Associations between neighborhood percent black residents and dialysis facility characteristics

Twenty-four dialysis facilities were identified, of which 19 (79.1%) were “for profit”. The median hospitalization rate reported by the dialysis facilities to CMS was 1.91 hospitalizations per patient per year (IQR 1.61–2.80) in 2016 (ED visit data were not available). The median Five Star rating of the dialysis facilities was 3 (IQR 3–4). There were no statistically significant associations between percent Black residents, percent of population living under the poverty line, and percent of residents that graduated high school of the block group of each dialysis facility and CMS reported hospitalization outcomes (*p* = 0.52, 0.49 and 0.80, respectively). Nor was there any association between percent Black residents, percent of population living under the poverty line, and percent of residents that graduated high school of the block group of each dialysis facility and their Five Star rating (*p* = 0.25, 0.17, 0.36 respectively).

## Discussion

Our study of a cohort of patients on hemodialysis who had at least one ED visit to a large hospital system showed a median IRR of 3 repeat ED visits during the study period. Living in block groups in the highest quintiles of percent Black residents was associated with an IRR of 4 avoidable ED visits. Hispanic and White subjects that lived in areas with the highest percentage of Black residents had higher IRR of avoidable ED visits than the Black residents, as did male patients on hemodialysis living in areas with the highest percentage of Black residents as compared to female patients. We found that being younger, a non-English speaker, and having a history of heart failure (most likely a proxy for episodes of fluid overload) are also significantly associated with ED visit rates. These results are consistent with other studies in the literature [30, 31]. The IRR for ED visits in our study is comparable to national rates, and the differential risks defined by neighborhood of residence is consistent with associations reported between neighborhood of residence and hospitalization outcomes in the ESKD population [4, 5, 27].

The history of racial segregation in America has led to the establishment of what we know today as residentially segregated metropolitan areas and was driven historically by systematic exclusion of Blacks from the two of the most generous and foundational programs for creating wealth and opportunity in the nation, the Homeowners’ Loan Act of 1933 which created the Home Owners Loan Corporation (HOLC) and the Servicemen’s Readjustment Act of 1944 or GI Bill which supported housing and education [32]. The persistent exclusion beyond civil rights legislation through exclusionary policies and/or practices have maintained segregation and perpetuated socioeconomic and other structural disparities that disproportionately affect predominantly Black neighborhoods [32]. Some contributors to neighborhood level health disparities are high crime rates, limited mobility, low quality schools, sub-standard housing, few recreational facilities and lack of wholesome food options, all of which are compounded by the constant financial and psychological stress of a lower class lifestyle [1]. Patients with ESKD are disproportionately clustered in large metropolitan, predominantly black neighborhoods [15].

An analysis by Rodriguez et al. found White and Black patients on hemodialysis that lived in predominantly Black neighborhoods had worse quality dialysis and longer times to transplant as compared to those who resided in zip codes with lower percentage of Blacks [16]. The mortality rates were higher in the White patients that lived in predominantly Black neighborhoods, as compared to Black patients [16]. Yan et al. showed lowest pre-ESKD nephrology care and arterio-venous fistula placement for young Blacks in metropolitan areas [17]. Within the first year of starting hemodialysis, adjusted cardiovascular and infection related hospitalizations were higher in younger Black and Hispanic patients [33]. In these studies, those neighborhoods with a higher percentage of Black residents also had lower median incomes, a higher percentage of families living under the poverty line and a lower percentage of residents with high school diplomas [3, 16, 33]. By contrast, our analysis of those block groups in the Bronx with the higher percentage of Black residents found fewer families living under the poverty line and with a higher percentage of residents having graduated high school. Our observations highlight demographic differences within racially clustered communities but also across different geographic areas. The finding that despite higher SES those living in predominantly Black neighborhoods had higher ED visit counts could be due to distrust of the medical establishment that has been reported to influence patients’ beliefs and behaviors for seeking and/or receiving appropriate care, and ability to achieve equitable clinical outcomes [34, 35]. Another factor could be stereotype threat, a more recently recognized concept in healthcare, that refers to the fear of being judged by, and/or of personally confirming through one’s own actions, negative group stereotypes that specifically operate within the domain of healthcare, such as inferior intelligence, lower status, and being less deserving of the highest standard of care, and has been identified to operate in older African Americans [36]. Furthermore, the finding that White and Hispanic patients from neighborhoods with a higher percentage of Black residents were at an even higher risk for avoidable ED visits than Black patients is consistent with social theories that explain the disproportionately bad health outcomes in these populations: positing that Whites and Hispanics who reside in predominantly Black neighborhoods are even more vulnerable than their Black counterparts [5]. Our finding that male patients on hemodialysis are at higher risk for avoidable ED visits in those neighborhoods with a higher percentage of Black residents supports prior data that shows that young Black men are less likely to access primary care and that those on hemodialysis are at higher risk for mortality than their counterparts [10, 37, 38] (Fig. 2).

Limitations of our study include the retrospective nature of the analysis, missing data for some of the clinical variables, as well as the lack of data with respect to dialysis vintage, although there is no reason to suspect that these phenomena should introduce differential bias into the interpretation of results that are based on neighborhood racial makeup. Our cohort excluded patients that never made an ED visit over the study period but previous work shows a high rate of ED visits in this population, therefore we feel comfortable that our sample represents most of the hemodialysis population served by our hospitals [22]. Though Montefiore is the predominant hospital in the Bronx and the fidelity of patients to the location of their recurrent ED visits is high, there is a possibility that a number of patients made ED visits to other hospitals in the Bronx and thus were not taken into account. This may introduce differential bias because two of the non-Montefiore hospitals are located in the South Bronx, which has a significantly socioeconomically disadvantaged population, by Bronx standards. However, because of the lower geographic variation in access to care and the availability of outpatient services by the one hospital system studied, residual confounding related to these variables is minimized. Finally we were unable to capture the laboratory data routinely collected at the outpatient dialysis facilities which would have been helpful in interpreting some of this data. Strengths of our analysis included minimal residual confounding related to differences in geographic access to care, hospital related services, and chronic illnesses requiring repeat hospitalizations (as we chose ED visits, with more of an emphasis on patient behavior, family/social support and care coordination, than hospitalizations).

## Conclusions

In conclusion, our study of a cohort of patients on hemodialysis in the Bronx shows that living in a neighborhood with a higher percentage of Blacks is associated with repeat ED visits, especially in males and non-Black patients (Hispanic and White). The implications of these findings support the role of structural and institutional barriers to effective outpatient care for the ESKD population and the fact that they disproportionately affect males living in predominantly Black neighborhoods. In order to mitigate inpatient resource utilization, attention should be paid to upstream barriers to effective outpatient care in predominantly Black neighborhoods and recognize that this affects patients of all backgrounds that live in these communities.

## Additional file

Additional file 1: CLG comorbidity materials. Ad hoc report comorbidity data. The PDF file serves as a reference for the derivation of the comorbidity index score. This was developed by Clinical Looking Glass to serve as an aide and reference for the derivations of the Charlson comorbidity score reported by Looking Glass. (PDF 1315 kb)

## Data Availability

The study database can be made available upon request.

## Abbreviations

ACS: American Community Survey
ADI: Area deprivation index
BMI: Body mass index
CLG: Clinical Looking Glass
CMS: Centers for Medicare Services
ED: Emergency department
ESKD: End Stage Kidney Disease
Hgb: Hemoglobin
ICD: International classification of disease
IQR: Interquartile range
IRR: Incident rate ratio
PD: Peritoneal dialysis
Permcath: Permanent catheter
SES: Socio-economic status
SNF: Skilled nursing facility
US: United States
