# Supplementary material for: The association of neighborhood racial mix and ED visit count in a cohort of patients on hemodialysis
Source: BMC Nephrol. 2019 Sep 2;20:343. doi: 10.1186/s12882-019-1520-x (PMC6720403; doi:10.1186/s12882-019-1520-x)
Supplement: Supplementary file 1 — CLG comorbidity materials. Ad hoc report comorbidity data. The PDF file serves as a reference for the derivation of the comorbidity index score. This was developed by Clinical Looking Glass to serve as an aide and reference for the derivations of the Charlson comorbidity score reported by Looking Glass. (PDF 1315 kb) [file 12882_2019_1520_MOESM1_ESM.pdf]

Chapter 11: Comorbidity "Sickliness Adjustment"

Background

In observational studies, when comparing outcomes in two cohorts of patients, analysts often wonder whether the compared populations were comparable at baseline. It is often asked colloquially have we controlled for "sickliness of our patients". "My patients are sicker than his patients". This sometimes is translated as "have we controlled for comorbidity"? Have we controlled for "severity of illness"?

Controlling for severity of illness implies that there exists an evaluation test that can be applied to a patient to extract some notion of how sick he is at this time. Such a magical metric does not exist. What has historically been done, has been to look at the last hospitalization, extract the ICD-9 diagnostic codes and develop a model that captures a notion of severity. Some have extended this approach to cover clinical variables extracted from the index hospitalization. **Comorbidity 1** will provide the user with a standard method for using a single hospitalization to capture severity of illness. The multiple ICD-9 will be summarized in a smaller set and in the case of the Charlson Index a specific value will summarize probability of mortality. It will also provide a summary of laboratory and clinical variables available from the index hospitalization to allow the user to build his own metric. The input for Comorbidity I will be the cohort whose index date is captured within the date range of date of admission and date of discharge of a hospitalization. Within includes the date of admission and date of discharge.

Comorbidity II, applies the same methodology over a duration of time capturing data from multiple hospitalizations, Clinic visits, and ED visits. This latter approach while not validated follows logically in the same spirit of Comorbidity I and allows the user to build his own metric.

Historical Approaches to "Sickliness Adjustment" - the Hazards of adjustment

Adjustment strategies in principle are dependant upon what you are trying to adjust for.

- Are you adjusting for severity of a specific disease so as to compare costs in populations with that disease to evaluate the utility of management strategies?
- Are you adjusting for the likelihood of mortality? If you are describing different diseases, the likelihood of mortality in disease A severity level severe might be much less than disease B with severity level moderate if the base mortality rate of B is greater than A.
- Are you adjusting for cost? The probability of high cost in one patient population might not be proportional to the probability of death as in some populations high likelihood of death would preclude using high cost interventions.
- Are you adjusting for likelihood of readmission? Likelihood of death might compete with likelihood of readmission.
- Are you just trying to get a description of the population in cogent categories and let your readers decide whether the populations are reasonably comparable at baseline?
- Are you trying to categorically describe each patient in a cohort in a limited number of cogent categories and then build a statistical model yourself using outcomes in your own populations to standardize the outcome in both populations (Elixhauser approach of AHRQ below).
- Are you trying to turn the "sickliness" question into a simple numeric summary so you can compare the "sickliness of the two populations"? This is the Charlson index approach that generates a Charlson score "predictive of mortality due to comorbid diseases" and is used to describe the "sickliness of two populations of patients". To what extent "probability of death" even if perfectly predicted by the Charlson index actually gets to the core of "sickliness" for the particular issue under study is open for debate.

All the aforementioned techniques take advantage of billing coding to qualify the patient population. All suffer from the bias that as billing coding changes

or the vigor with which they are recorded changes, the "apparent" sickliness of the patients change.

Also, any index developed can not take into consideration changes in technology and therefore the clinical consequences of a particular diagnosis. A Charlson score for AIDS at 6 developed in 1987, does not take into consideration the major advances in HIV care over the last few years.

Any index must be validated. As coding practices change, as new ICD-9 codes are included validation studies do not keep up with the changing nomenclature. AHRQ, has a website that identifies the new icd-9 codes and assigns them to the Elixhauser comorbidity categories. While these assignments may have face validity, there is no formal process for revalidating how these new mappings will behave in predictive models. It is possible that the new codes might be better segregated into a different category rather than subsumed in one with the suspected same face validity.

When new indices are developed based upon ICD-9 categories that did not exist in previous years, the ability to perform adjusted "sickliness" studies across time may have some validity problems. This is inherent to the changing nomenclature and understanding of disease and impacts upon both the "open source adjusters" and commercially available adjusters.

APR DRG

Commercially available adjusters for mortality include the 3M apr-drg. The apr drg develops drgs and develops severity levels within drg based upon comorbid diseases and then assigns a weight to the statistical model to predict death during hospitalization. It is a death predictor and its data base of hundreds of thousands of patients allows it to develop coefficients for each APR DRG cluster. The 3m apr drg creates for each hospital patient and then for the aggregate of patients in that hospital a predicted mortality rate based upon the diagnoses of the patients and compares this result to the actual observed deaths. How well the weight of probability of death is useful as an adjuster for other issues other than death is not clear.

Apr DRG has been developed in cooperation with a children's hospital so its coverage of infants and children is much better than other adjusters.

Federal DRG Weight

The Federal government assigns DRGs mapped from ICD-9 codes. The mapping changes annually, but in 2007, there was a complete renaming of the DRG codes. In addition, annually, the Federal Government to achieve net cost neutrality assign Weights (important to reimbursement) to each of the DRG categories. The Weights are suppose to reflect the cost in caring for the patients in those categories and in principle are to reflect the relative reimbursement that should be recognized for each DRG. In a sense, this "cost weight" identifies what legitimate burden of effort each patient requires relative to the effort expended in all hospitalized patients. This DRG weight can be seen as an adjuster for disease burden to the extent that you believe that federal recognition of effort required is related in some measure to sickliness. Of course, there are some people who cost a lot but may not have a higher propensity for the specific outcome of interest.

Federal Weights are recalibrated annually so if you wish to compare across time you would need to apply the DRG grouper of the most recent year across time and accept the Fed Weight of the most recent time period as the appropriate Fed Weight. The problem with this solution using a common grouper and weight system across time is that the DRG grouper depends upon the existence of specific ICD-9 in the vocabulary. If in previous years that ICD-9 did not exist, the grouper can not credit you for that ICD-9. Moreover, in recent years there has been a weighting penalty for less specific diagnoses so that if you coded CHF nonspecific you would not be as rewarded financially as you would have been by coding "CHF diastolic dysfunction". Present groupers reward specificity. This specificity of diagnosis was not present in previous years, causing a present day grouper applied on the past to potentially under weight the patients from the past in a study comparing patient outcomes across time.

In the future, we will provide the user an option to either use the weight from a common drg grouper across time or use the Fed DRG weight that would have been generated by the then applicable Grouper in the year of interest.

Another weakness of Federal Weights is that that it is focussed on the Medicare population and provides much more limited information in children or infants.

Problem with all the diagnosis based Adjusters

All of these methodologies suffer from the problem that if you are trying to adjust for comorbidity to create a metric that looks at clinical quality but you use an adjuster that forgives outcome by extra diagnoses that could be the result of poor medical care, you have decreased the power of the instrument to detect clinical incompetence. The most clinically incompetent institution will generate the greatest number of diagnoses because their errors cause infection and side effects that are then used by the severity adjuster to "forgive" their higher mortality rate.

The use of race in these models also suffers from the possibility that poor care provided disproportionately to people of a particular race (race as a sociologic concept), is "forgiven" when race is included in the statistical "adjusting model".

Clinical Data for Adjusters

Clinical Data has been incorporated in formal ICU mortality severity adjusters in

- Apache IV (Adult ICU patients)
- Prism (neonatal icu)

Researchers performing readmission analyses and Length of Stay analyses have found that the inclusion of albumin or other laboratory data elements often improves the performance of the predictive models that otherwise include only administrative data.

We provide options for obtaining laboratory data and non laboratory covariates to build adjustment models.

There are no perfect answers. This smart report provides the user with two conventional approaches in using administrative data for "adjustment purposes".

Algorithm

References:

1. Quan et al. Coding algorithms for defining comorbidities in ICD-9-CM and ICD-10 administrative data. Med Care 2005 Nov; 43(11):1073-1077

2. Charlson et al. A New Method of Classifying Prognostic Comorbidity in Longitudinal Studies:Development and Validation J Chron Dis 1987 ;40(3); 373-383

3. Elixhauser Ahrq Web ICD-9 but enhanced with but enhanced to reflect ahrq comorbidity calculator through FY 2007. comorbidity software version 3.2
   a. http://www.hcup-us.ahrq.gov/toolssoftware/comorbidity/Table1-FY2007-V3_2.pdf and
   b. http://www.hcup-us.ahrq.gov/toolssoftware/comorbidity/Table1-FY2006-V3_1.pdf

4. Hutchinson TA Thomas DC MacGibbon B. Predicting survival in adults with end-stage renal disease: An age equivalence index. Ann Intern Med. 1982; 96: 417-423.

5. Charlson Enhanced Comorbidity ICD-9. ref 1,2 Table 1 (enhanced ICD-9 cm)
6. Elixhauser AHRQ Web ICD-9-CM.
   ref 1, Table 2 (third column), FY '06, FY '07 updates (reference 3)
7. Elixhauser Enhanced ICD-9-CM
   reference 1, Table 2 third column no web updates

Charlson Comorbidity Comorbidity Categories and Index

The ICD-9’s of a specific hospitalization are mapped to specific categories in the Charlson comorbidity index. The report provides an excel column for each of these categories signifying with a “1” the presence of this category and with a “0” the absence of this category. The report also provides a weighted score:

(A web version of this algorithm is available at: http://www.medalreg.com/qhc/medal/ch1/1_13/01-13-01-ver9.php3 see info button content below)

Weighted Index of Comorbidity:

**Condition**	**Assigned Weight**
Myocardial Infarction	1
Congestive Heart Failure	1
Peripheral Vascular Disease	1
Cerebrovascular Disease	1
Dementia	1
Chronic Pulmonary Disease	1
Connective Tissue Disease	1
Ulcer Disease	1
Liver Disease Mild	1
Diabetes	1
Hemiplegia	2
Renal Disease Moderate or Severe	2
Diabetes with End Organ Damage	2
Any Malignancy	2
Leukemia	2
Malignant Lymphoma	2
Liver Disease. Moderate or Severe	3
Metastatic Solid Malignancy	6

AIDS 6

The impact of age is calculated separately with the following scores:
less than 50 scored 0
50-59 scored 1
60-69 scored 2
70-79 scored 3
80-89 scored 4
(and continuing with each decade)

age-related risk =
= INT((age-40) / 10)

A probability of ten year survival:
combined score =
= (weighted index of comorbidity) + (age-related risk)

estimated 10 year survival =
= 0.983 EXP (EXP(0.9 * (combined score)))
where:
• 0.983 is the 10 year survival in a low risk population (from Hutchinson et al 1982 reference

Note: Charlson in Comorbidity 1 looks at diagnoses in a single hospitalization. As a result you never see a coding situation where both diabetes with and diabetes without complications would exist in a single hospitalization. Similarly, liver disease is either mild, moderate, or severe in a single hospitalization. In Comorbidity 2, the unvalidated extension of the Charlson, we may have over an interval of time both diabetes designations or both liver designations. The rule is to count only the most severe one in the interval for the purpose of calculating a Charlson score. The Charlson weight becomes:

If diabetes no complication then 1
if diabetes complications then 2
if both then 2

if liver disease mild 1
if liver disease moderate or severe 3
if both 3

if moderate or severe renal disease 2

Table 1 Enhanced ICD-9-CM Charlson Comordítity Criteria (column 4)

TABLE 1. ICD-9-CM and ICD-10 Coding Algorithms for Charlson Comorbidities

Comorbidities	Deyo's ICD-9-CM	ICD-10	Enhanced ICD-9-CM
Myocardial infarction	410.x, 412.x	I21.x, I22.x, I25.2	410.x, 412.x
Congestive heart failure	428.x	I09.9, I11.0, I13.0, I13.2, I25.5, I42.0, I42.5–I42.9, I43.x, I50.x, P29.0	398.91, 402.01, 402.11, 402.91, 404.01, 404.03, 404.11, 404.13, 404.91, 404.93, 425.4–425.9, 428.x
Peripheral vascular disease	443.9, 441.x, 785.4, V43.4 Procedure 38.48	I70.x, I71.x, I73.1, I73.8, I73.9, I77.1, I79.0, I79.2, K55.1, K55.8, K55.9, Z95.8, Z95.9	093.0, 437.3, 440.x, 441.x, 443.1–443.9, 47.1, 557.1, 557.9, V43.4
Cerebrovascular disease	430.x–438.x	G45.x, G46.x, H34.0, I60.x–I69.x	362.34, 430.x–438.x
Dementia	290.x	F00.x–F03.x, F05.1, G30.x, G31.1	290.x, 294.1, 331.2
Chronic pulmonary disease	490.x–505.x, 506.4	I27.8, I27.9, J40.x–J47.x, J60.x–J67.x, J68.4, J70.1, J70.3	416.8, 416.9, 490.x–505.x, 506.4, 508.1, 508.8
Rheumatic disease	710.0, 710.1, 710.4, 714.0–714.2, 714.81, 725.x	M05.x, M06.x, M31.5, M32.x–M34.x, M35.1, M35.3, M36.0	446.5, 710.0–710.4, 714.0–714.2, 714.8, 725.x
Peptic ulcer disease	531.x–534.x	K25.x–K28.x	531.x–534.x
Mild liver disease	571.2, 571.4–571.6	B18.x, K70.0–K70.3, K70.9, K71.3–K71.5, K71.7, K73.x, K74.x, K76.0, K76.2–K76.4, K76.8, K76.9, Z94.4	070.22, 070.23, 070.32, 070.33, 070.44, 070.54, 070.6, 070.9, 570.x, 571.x, 573.3, 573.4, 573.8, 573.9, V42.7
Diabetes without chronic complication	250.0–250.3, 250.7	E10.0, E10.1, E10.6, E10.8, E10.9, E11.0, E11.1, E11.6, E11.8, E11.9, E12.0, E12.1, E12.6, E12.8, E12.9, E13.0, E13.1, E13.6, E13.8, E13.9, E14.0, E14.1, E14.6, E14.8, E14.9	250.0–250.3, 250.8, 250.9
Diabetes with chronic complication	250.4–250.6	E10.2–E10.5, E10.7, E11.2–E11.5, E11.7, E12.2–E12.5, E12.7, E13.2–E13.5, E13.7, E14.2–E14.5, E14.7	250.4–250.7
Hemiplegia or paraplegia	344.1, 342.x	G04.1, G11.4, G80.1, G80.2, G81.x, G82.x, G83.0–G83.4, G83.9	334.1, 342.x, 343.x, 344.0–344.6, 344.9
Renal disease	582.x, 583–583.7, 585.x, 586.x, 588.x	I12.0, I13.1, N03.2–N03.7, N05.2–N05.7, N18.x, N19.x, N25.0, Z49.0–Z49.2, Z94.0, Z99.2	403.01, 403.11, 403.91, 404.02, 404.03, 404.12, 404.13, 404.92, 404.93, 582.x, 583.0–583.7, 585.x, 586.x, 588.0, V42.0, V45.1, V56.x
Any malignancy, including lymphoma and leukemia, except malignant neoplasm of skin	140.x–172.x, 174.x–195.8, 200.x–208.x	C00.x–C26.x, C30.x–C34.x, C37.x–C41.x, C43.x, C45.x–C58.x, C60.x–C76.x, C81.x–C85.x, C88.x, C90.x–C97.x	140.x–172.x, 174.x–195.8, 200.x–208.x, 238.6
Moderate or severe liver disease	456.0–456.21, 572.2–572.8	I85.0, I85.9, I86.4, I98.2, K70.4, K71.1, K72.1, K72.9, K76.5, K76.6, K76.7	456.0–456.2, 572.2–572.8
Metastatic solid tumor	196.x–199.1	C77.x–C80.x	196.x–199.x
AIDS/HIV	042.x–044.x	B20.x–B22.x, B24.x	042.x–044.x

Figure 11-1: *Enhanced Charlson Comorbidity Criteria*

TABLE 2. ICD-9-CM and ICD-10 Coding Algorithms for Elixhauser Comorbidities

Comorbidities	Elixhauser's Original ICD-9-CM	Elixhauser AHRQ-Web ICD-9-CM	ICD-10	Enhanced ICD-9-CM
Congestive heart failure	398.91, 402.11, 402.91, 404.11, 404.13, 404.91, 404.93, 428.x	398.91, 402.01, 402.11, 402.91, 404.01, 404.03, 404.11, 404.13, 404.91, 404.93, 428.x	I09.9, I11.0, I13.0, I13.2, I25.5, I42.0, I42.5–I42.9, I43.x, I50.x, P29.0	398.91, 402.01, 402.11, 402.91, 404.01, 404.03, 404.11, 404.13, 404.91, 404.93, 425.4–425.9, 428.x
Cardiac arrhythmias	426.10, 426.11, 426.13, 426.2–426.53, 426.6–426.8, 427.0, 427.2, 427.31, 427.60, 427.9, 785.0, V45.0, V53.3	—	I44.1–I44.3, I45.6, I45.9, I47.x–I49.x, R00.0, R00.1, R00.8, T82.1, Z45.0, Z95.0	426.0, 426.13, 426.7, 426.9, 426.10, 426.12, 427.0–427.4, 427.6–427.9, 785.0, 996.01, 996.04, V45.0, V53.3
Valvular disease	093.2, 394.0–397.1, 424.0–424.91, 746.3–746.6, V42.2, V43.3	093.2, 394.x–397.1, 397.9, 424.x, 746.3–746.6, V42.2, V43.3	A52.0, I05.x–I08.x, I09.1, I09.8, I34.x–I39.x, Q23.0–Q23.3, Z95.2–Z95.4	093.2, 394.x–397.x, 424.x, 746.3–746.6, V42.2, V43.3
Pulmonary circulation disorders	416.x, 417.9	416.x, 417.9	I26.x, I27.x, I28.0, I28.8, I28.9	415.0, 415.1, 416.x, 417.0, 417.8, 417.9
Peripheral vascular disorders	440.x, 441.2, 441.4, 441.7, 441.9, 443.1–443.9, 447.1, 557.1, 557.9, V43.4	440. x, 441.x, 442.x, 443.1–443.9, 447.1, 557.1, 557.9, V43.4	I70.x, I71.x, I73.1, I73.8, I73.9, I77.1, I79.0, I79.2, K55.1, K55.8, K55.9, Z95.8, Z95.9	093.0, 437.3, 440.x, 441.x, 443.1– 443.9, 447.1, 557.1, 557.9, V43.4
Hypertension, uncomplicated	401.1, 401.9	401.1, 401.9, 642.0	I10.x	401.x
Hypertension, complicated	402.10, 402.90, 404.10, 404.90, 405.1, 405.9	401.0, 402.x–405.x, 642.1, 642.2, 642.7, 642.9	I11.x–I13.x, I15.x	402.x–405.x
Paralysis	342.0, 342.1, 342.9–344.x	342.x–344.x, 438.2–438.5	G04.1, G11.4, G80.1, G80.2, G81.x, G82.x, G83.0–G83.4, G83.9	334.1, 342.x, 343.x, 344.0–344.6, 344.9
Other neurological disorders	331.9, 332.0, 333.4, 333.5, 334.x, 335.x, 340.x, 341.1–341.9, 345.0, 345.1, 345.4, 345.5, 345.8, 345.9, 348.1, 348.3, 780.3, 784.3	330.x–331.x, 332.0, 333.4, 333.5, 334.x–335.x, 340, 341.1–341.9, 345.x, 347.x, 780.3, 784.3	G10.x–G13.x, G20.x–G22.x, G25.4, G25.5, G31.2, G31.8, G31.9, G32.x, G35.x–G37.x, G40.x, G41.x, G93.1, G93.4, R47.0, R56.x	331.9, 332.0, 332.1, 333.4, 333.5, 333.92, 334.x–335.x, 336.2, 340.x, 341.x, 345.x, 348.1, 348.3, 780.3, 784.3
Chronic pulmonary disease	490–492.8, 493.00–493.91, 494.x–505.x, 506.4	490.x–492.x, 493.x, 494.x–505.x, 506.4	I27.8, I27.9, J40.x–J47.x, J60.x–J67.x, J68.4, J70.1, J70.3	416.8, 416.9, 490.x –505.x, 506.4, 508.1, 508.8
Diabetes, uncomplicated	250.0–250.3	250.0–250.3, 648.0	E10.0, E10.1, E10.9, E11.0, E11.1, E11.9, E12.0, E12.1, E12.9, E13.0, E13.1, E13.9, E14.0, E14.1, E14.9	250.0–250.3
Diabetes, complicated	250.4–250.7, 250.9	250.4–250.9, 775.1	E10.2–E10.8, E11.2–E11.8, E12.2–E12.8, E13.2–E13.8, E14.2–E14.8	250.4–250.9
Hypothyroidism	243–244.2, 244.8, 244.9	243–244.2, 244.8, 244.9	E00.x–E03.x, E89.0	240.9, 243.x, 244.x, 246.1, 246.8
Renal failure	403.11, 403.91, 404.12, 404.92, 585.x, 586.x, V42.0, V45.1, V56.0, V56.8	403.01, 403.11, 403.91, 404.02, 404.03, 404.12, 404.13, 404.92, 404.93, 585.x, 586.x, V42.0, V45.1, V56.x	I12.0, I13.1, N18.x, N19.x, N25.0, Z49.0–Z49.2, Z94.0, Z99.2	403.01, 403.11, 403.91, 404.02, 404.03, 404.12, 404.13, 404.92, 404.93, 585.x, 586.x, 588.0, V42.0, V45.1, V56.x
Liver disease	070.32, 070.33, 070.54, 456.0, 456.1, 456.2, 571.0, 571.2–571.9, 572.3, 572.8, V42.7	070.22, 070.23, 070.32, 070.33, 070.44, 070.54, 456.0, 456.1, 456.20, 571.0, 571.2–571.9, 572.3, 572.8, V42.7	B18.x, I85.x, I86.4, I98.2, K70.x, K71.1, K71.3–K71.5, K71.7, K72.x–K74.x, K76.0, K76.2–K76.9, Z94.4	070.22, 070.23, 070.32, 070.33, 070.44, 070.54, 070.6, 070.9, 456.0–456.2, 570.x, 571.x, 572.2–572.8, 573.3, 573.4, 573.8, 573.9, V42.7

(Continued)

Figure 11-2: *Elixhauser Web ICD-9 CM web (third column of table 2 further enhanced by updates that follow)*

TABLE 2. *(Continued)*

Comorbidities	Elixhauser's original ICD-9-CM	Elixhauser AHRQ-Web ICD-9-CM	ICD-10	Enhanced IC-9-CM
Peptic ulcer disease excluding bleeding	531.70, 531.90, 532.70, 532.90, 533.70, 533.90, 534.70, 534.90, V12.71	531.41, 531.51, 531.61, 531.7, 531.91, 532.41, 532.51, 532.61, 532.7, 532.91, 533.41, 533.51, 533.61, 533.7, 533.91, 534.41, 534.51, 534.61, 534.7, 534.91	K25.7, K25.9, K26.7, K26.9, K27.7, K27.9, K28.7, K28.9	531.7, 531.9, 532.7, 532.9, 533.7, 533.9, 534.7, 534.9
AIDS/HIV	042.x–044.x	042.x–044.x	B20.x–B22.x, B24.x	042.x–044.x
Lymphoma	200.x–202.3x, 202.5–203.0, 203.8, 238.6, 273.3, V10.71, V10.72, V10.79	200.x–202.3, 202.5–203.0, 203.8, 238.6, 273.3	C81.x–C85.x, C88.x, C96.x, C90.0, C90.2	200.x–202.x, 203.0, 238.6
Metastatic cancer	196.x–199.x	196.x–199.x	C77.x–C80.x	196.x–199.x
Solid tumor without metastasis	140.x–172.x, 174.x, 175.x, 179.x–195.x, V10.x	140.x–172.x, 174.x, 175.x, 179.x–195.x	C00.x–C26.x, C30.x–C34.x, C37.x–C41.x, C43.x, C45.x–C58.x, C60.x–C76.x, C97.x	140.x–172.x, 174.x–195.x
Rheumatoid arthritis/ collagen vascular diseases	701.0, 710.x, 714.x, 720.x, 725.x	701.0, 710.x, 714.x, 720.x, 725.x	L94.0, L94.1, L94.3, M05.x, M06.x, M08.x, M12.0, M12.3, M30.x, M31.0–M31.3, M32.x–M35.x, M45.x, M46.1, M46.8, M46.9	446.x, 701.0, 710.0–710.4, 710.8, 710.9, 711.2, 714.x, 719.3, 720.x, 725.x, 728.5, 728.89, 729.30
Coagulopathy	286.x, 287.1, 287.3–287.5	286.x, 287.1, 287.3–287.5	D65–D68.x, D69.1, D69.3–D69.6	286.x, 287.1, 287.3–287.5
Obesity	278.0	278.0	E66.x	278.0
Weight loss	260.x–263.x	260.x–263.x, 783.2	E40.x–E46.x, R63.4, R64	260.x–263.x, 783.2, 799.4
Fluid and electrolyte disorders	276.x	276.x	E22.2, E86.x, E87.x	253.6, 276.x
Blood loss anemia	280.0	280.0, 648.2	D50.0	280.0
Deficiency anemia	280.1–281.9, 285.9	280.1–281.9, 285.2, 285.9	D50.8, D50.9, D51.x–D53.x	280.1–280.9, 281.x
Alcohol abuse	291.1, 291.2, 291.5–291.9, 303.9, 305.0, V113	291.0–291.3, 291.5, 291.8, 291.9, 303.x, 305.0	F10, E52, G62.1, I42.6, K29.2, K70.0, K70.3, K70.9, T51.x, Z50.2, Z71.4, Z72.1	265.2, 291.1–291.3, 291.5–291.9, 303.0, 303.9, 305.0, 357.5, 425.5, 535.3, 571.0–571.3, 980.x, V11.3
Drug abuse	292.0, 292.82–292.89, 292.9, 304.0, 305.2–305.9	292.0, 292.82–292.89, 292.9, 304.x, 305.2–305.9, 648.3	F11.x–F16.x, F18.x, F19.x, Z71.5, Z72.2	292.x, 304.x, 305.2–305.9, V65.42
Psychoses	295.x–298.x, 299.1	295.x–298.x, 299.1	F20.x, F22.x–F25.x, F28.x, F29.x, F30.2, F31.2, F31.5	293.8, 295.x, 296.04, 296.14, 296.44, 296.54, 297.x, 298.x
Depression	300.4, 301.12, 309.0, 309.1, 311	300.4, 301.12, 309.0, 309.1, 311	F20.4, F31.3–F31.5, F32.x, F33.x, F34.1, F41.2, F43.2	296.2, 296.3, 296.5, 300.4, 309.x, 311

Figure 11-3: *Elixhauser Web ICD-9 CM web (third column of table 2 further enhanced by updates that follow)*

Additions to Elixhauser to achieve compatibility with comorbidity version 3.2

FY 2006 enhancements- http://www.hcup-us.ahrq.gov/toolssoftware/comorbidity/Table1-FY2006-V3_1.pdf

(*Table is on the next page*)

ICD-9-CM Changes to the Comorbidity Software		
ICD-9-CM Diagnosis Code	ICD-9-CM Diagnosis Code Label	Comorbidity Assignment
4372	Hypertensive Encephalopathy	Hypertension - complicated
*5853	Chronic kidney disease Stage III (moderate)	Renal failure
*5854	Chronic kidney disease Stage IV (severe)	Renal failure
*5855	Chronic kidney disease Stage V	Renal failure
*5856	End stage renal disease	Renal failure
*5859	Chronic kidney disease unspec	Renal failure
*29182	Alcohol induced sleep disorders	Alcohol abuse
3337	Acquired torsion dystonia	Other neurological disorders
78031	Febrile convulsions (simple), unspecified	Other neurological disorders

Figure 11-4: *ICD-9-CM changes to the comorbidity software FY 2006*

FY 2007 enhancements- http://www.hcup-us.ahrq.gov/toolssoftware/comorbidity/Table1-FY2007-V3_2.pdf

(*Table is on the next page*)

Table 1. Changes Made to Comorbidity Software for FY2007, Version 3.2

The following changes were made to the Comorbidity Software for fiscal year 2007. This year, ICD-9-CM and DRG updates for fiscal year 2007 are included. These changes are incorporated in the tool currently available: Comorbidity Software, Version 3.2.

ICD-9-CM Changes to the Comorbidity Software

ICD-9-CM Diagnosis Code	ICD-9-CM Diagnosis Code Label	Comorbidity Assignment
*333.71	Athetoid cerebral palsy	Other neurological disorders
*333.72	Acute dystonia due to drugs	Other neurological disorders
*333.79	Other acquired torsion dystonia	Other neurological disorders
*333.85	Subacute dyskinesia due to drugs	Other neurological disorders
*333.94	Restless legs syndrome [RLS]	Other neurological disorders
*338.0	Central pain syndrome	Other neurological disorders
*649.10	Obesity comp preg, birth, or puerperium, unspecified episode of care	Obesity
*649.11	Obesity comp preg, birth, or puerperium, delivered with antepartum complication	Obesity
*649.12	Obesity comp preg, delivered with postpartum complication	Obesity
*649.13	Obesity comp preg, antepartum condition or complication	Obesity
*649.14	Obesity comp preg, birth, or puerperium, postpartum cond/comp	Obesity
*649.30	Coagulation defects comp preg, birth, or the puerperium, unspec episode of care	Coagulation deficiency
*649.31	Coagulation defects comp preg, delivered with antepartum complication	Coagulation deficiency
*649.32	Coagulation defects comp preg, delivered with postpartum complication	Coagulation deficiency
*649.33	Coagulation defects comp preg, antepartum condition or complication	Coagulation deficiency
*649.34	Coagulation defects comp preg, birth, or the puerperium, postpartum cond/comp	Coagulation deficiency
*649.40	Epilepsy comp preg, birth, or the puerperium, unspec episode of care	Other neurological disorders
*649.41	Epilepsy comp preg, delivered with antepartum complication	Other neurological disorders
*649.42	Epilepsy comp preg, delivered with postpartum complication	Other neurological disorders
*649.43	Epilepsy comp preg, antepartum condition or complication	Other neurological disorders
*649.44	Epilepsy comp preg, birth, or the puerperium, postpartum cond/comp	Other neurological disorders
*768.7	Hypoxic ischemic encephalopathy [HIE]	Other neurological disorders
*780.32	Complex febrile convulsions	Other neurological disorders
*780.97	Altered mental status	Other neurological disorders
*793.91	Image test inconclusive due to excess body fat	Obesity

Figure 11-5: *Changes to the Comorbidity Software for FY 2007*

Table 1. Changes Made to Comorbidity Software for FY2011, Version 3.6

The following changes were made to the Comorbidity Software for fiscal year 2011. This year includes ICD-9-CM updates. These changes are incorporated in the tool currently available: Comorbidity Software, Version 3.6.

ICD-9-CM Changes to the Comorbidity Software		
ICD-9-CM Diagnosis Code	ICD-9-CM Diagnosis Code Label	Comorbidity Assignment
*780.33	Post traumatic seizures	Other neurological disorders
*278.03	Obesity hypoventilation syndrome	Obesity
*V85.41	Body Mass Index 40.0-44.9, adult	Obesity
*V85.42	Body Mass Index 45.0-49.9, adult	Obesity
*V85.43	Body Mass Index 50.0-59.9, adult	Obesity
*V85.44	Body Mass Index 60.0-69.9, adult	Obesity
*V85.45	Body Mass Index 70 and over, adult	Obesity

*ICD-9-CM changes designated with an asterisk are new ICD-9-CM codes introduced in FY2011.

Current as of November 2010.

Figure 11-6 *Changes to the Comorbidity software for FY2011*

Table 1. Changes Made to Comorbidity Software for FY2012, Version 3.7

The following changes were made to the Comorbidity Software for fiscal year 2012. This year includes ICD-9-CM updates. These changes are incorporated in the tool currently available: Comorbidity Software, Version 3.7.

ICD-9-CM Changes to the Comorbidity Software		
ICD-9-CM Diagnosis Code	ICD-9-CM Diagnosis Code Label	Comorbidity Assignment
*415.13	Saddle embolus of pulmonary artery	Pulmonary Circulation disorders
*573.5	Hepatopulmonary syndrome	Liver disease
*286.52	Acquired hemophilia	Coagulation deficiency
*286.53	Antiphospholipid antibody with hemorrhagic disorder	Coagulation deficiency
*286.59	Other hemorrhagic disorder due to intrinsic circulating anticoagulants, antibodies, or inhibitors	Coagulation deficiency

*ICD-9-CM changes designated with an asterisk are new ICD-9-CM codes introduced in FY2012.

MS-DRG 252 was added to the Cardiac DRG exclusions as a correction.

Current as of October 2011.

Figure 11-7:*Changes to the Comorbidity software for FY2012*

Output

The output will include the following fields:

1. Medical Record Number
2. Home Phone
3. Patient Display name
4. Race
5. Ethnicity

6. Provider
7. Age at admission
8. Age in days at admission
9. Account ID
10. Admission Date
11. DRG ID (2007) - The federal drg grouper of October 2007 has been applied to the ICD-9 codes to generate a DRG ID number, DRG display name, and DRG weight.
12. DRG Weight (2007)
13. DRG Description (2007)
14. Charlson Categories follow in subsequent columns starting with myocardial infarction (1 = present 0= absent)
15. Congestive Heart Failure (1 = present 0= absent)
16. Peripheral Vascular Disease (1 = present 0= absent)
17. Cerebrovascular Disease (1 = present 0= absent)
18. Dementia (1 = present 0= absent)
19. Chronic Pulmonary Disease (1 = present 0= absent)
20. Rheumatologic Disease (1 = present 0= absent)
21. Peptic Ulcer Disease (1 = present 0= absent)
22. Mild Liver Disease (1 = present 0= absent)
23. Diabetes without complications (1 = present 0= absent)
24. Diabetes with Chronic Complications (1 = present 0= absent)
25. Hemiplegia or Paraplegia (1 = present 0= absent)
26. Renal Disease (1 = present 0= absent)
27. Any Malignancy (1 = present 0= absent)
28. Moderate or Severe Liver Disease (1 = present 0= absent)
29. Metastatic Solid Tumor (1 = present 0= absent)
30. AIDS (HIV) (1 = present 0= absent)
31. Charlson Score - the charlson score without age
32. Age Related - the contribution of Age to the Charlson score
33. Combined - the total score of Charlson plus age related
34. Estimated Percent 10 year survival - as indicated in the original paper

A sample comorbidity report:

DrgWeight	DrgDesc	Myocardial Infarction	Congestive Heart Failurevalues	Peripheral Vascular Disorders
0.8258	SEIZURES W/O MCC	0	0	0
0.6792	OTHER DISORDERS OF THE EYE W/O MCC	0	0	0
0.6792	OTHER DISORDERS OF THE EYE W/O MCC	0	0	0
1.7992	MAJOR HEAD & NECK PROCEDURES W CC/MCC OR MAJOR DEVICE	0	0	0
0.6227	EPISTAXIS W/O MCC	0	1	0
3.2677	OTHER RESP SYSTEM O.R. PROCEDURES W MCC	0	1	0

Figure 11-10: *First part of the report*

Metastatic Solid Tumor	AIDS/HIV	Charlson Score	Age Related Risk	Combined Score	Estimated % 10-yr survival
0	0	0	3	3	77.5
0	0	0	5	5	21.4
0	0	2	2	4	53.4
0	0	0	4	4	53.4
0	0	2	4	6	2.2
0	0	1	4	5	21.4
0	0	0	4	4	53.4

Figure 11-11: *Second part of the report*

Comorbidity Method Supplemental Information

Charlson Comorbidity Categories and Index

The ICD-9s and ICD-10s of a specific hospitalization are mapped to specific categories in the Charlson comorbidity index. The method output provides a column for each of these categories signifying with a "1" the presence of this category and with a "0" the absence of this category. The method output also provides a weighted score:

Weighted Index of Comorbidity:

Condition	Assigned Weight
Myocardial Infarction	1
Congestive Heart Failure	1
Peripheral Vascular Disease	1
Cerebrovascular Disease	1
Dementia	1
Chronic Pulmonary Disease	1
Rheumatic disease	1
Peptic Ulcer Disease	1
Mild Liver Disease	1
Diabetes without chronic complication	1
Hemiplegia or paraplegia	2
Renal Disease	2
Diabetes with chronic complication	2
Any malignancy, including lymphoma and leukemia, except malignant neoplasm of skin	2
Moderate or severe liver disease	3
Metastatic Solid Tumor	6
AIDS/HIV	6

The impact of age is calculated separately with the following scores:
less than 50 scored 0
50-59 scored 1
60-69 scored 2
70-79 scored 3

80-89 scored 4
(and continuing with each decade)

Age-related risk

= INT((age-40) / 10)

Combined score

= (weighted index of comorbidity) + (age-related risk)

Estimated ten year survival

= 0.983 EXP (EXP(0.9 * (combined score)))
where:
• 0.983 is the 10 year survival in a low risk population

Duplicate Designations

A patient may have over an interval of time both diabetes designations or both liver designations. The rule is to count only the most severe one in the interval for the purpose of calculating a Charlson score. The Charlson weight becomes:
If diabetes no complication then 1
if diabetes complications then 2
if both then 2
if liver disease mild 1
if liver disease moderate or severe 3
if both 3
if moderate or severe renal disease 2

TABLE 1. ICD-9-CM and ICD-10 Coding Algorithms for Charlson Comorbidities

Comorbidities	Deyo's ICD-9-CM +	ICD-10 *	Enhanced ICD-9-CM *
Myocardial infarction	410.x, 412.x	I21.x, I22.x, I25.2	410.x, 412.x
Congestive heart failure	428.x	I09.9,I11.0, I13.0, I13.2, I25.5, I42.0, I42.5-I42.9, I43.x, I50.x, P29.0	398.91, 402.01, 402.11, 402.91, 404.01, 404.03, 404.11, 404.13, 404.91, 404.93, 425.4-425.9, 428.x
Peripheral vascular disease	443.9, 441.x, 785.4, V43.4 Procedure 38.48	I70.x, I71.x, I73.1, I73.8, I73.9, I77.1, I79.0, I79.2, K55.1, K55.8, K55.9, Z95.8, Z95.9	093.0, 437.3, 440.x, 441.x, 443.1-443.9, 447.1, 557.1, 557.9, V43.4
Cerebrovascular disease	430.x-438.x	G45.x, G46.x, H34.0, I60.x-I69.x	362.34, 430.x-438.x
Dementia	290.x	F00.x-F03.x, F05.1, G30.x, G31.1	290.x, 294.1, 331.2
Chronic pulmonary disease	490.x-505.x, 506.4	I27.8, I27.9, J40.x-J47.x, J60.x-J67.x, J68.4, J70.1, J70.3	416.8, 416.9, 490.x-505.x, 506.4, 508.1, 508.8
Rheumatic disease	710.0, 710.1, 710.4, 714.0-714.2, 714.81, 725.x	M05.x, M06.x, M31.5, M32.x-M34.x, M35.1, M35.3, M36.0	446.5, 710.0-710.4, 714.0-714.2, 714.8, 725.x
Peptic ulcer disease	531.x-534.x	K25.x-K28.x	531.x-534.x
Mild liver disease	571.2, 571.4-571.6	B18.x, K70.0-K70.3, K70.9, K71.3-K71.5, K71.7, K73.x, K74.x, K76.0, K76.2-K76.4, K76.8, K76.9, Z94.4	070.22, 070.23, 070.32, 070.33, 070.44, 070.54, 070.6, 070.9, 570.x, 571.x, 573.3, 573.4, 573.8, 573.9, V42.7
Diabetes without chronic complication	250.0-250.3, 250.7	E10.0, E10.l, E10.6, E10.8, E10.9, E11.0, E11.1, E11.6, E11.8, E11.9, E12.0, E12.1, E12.6, E12.8, E12.9, E13.0, E13.1, E13.6, E13.8, E13.9, E14.0, E14.1, E14.6, E14.8, E14.9	250.0-250.3, 250.8, 250.9
Diabetes with chronic complication	250.4-250.6	E10.2-E10.5, E10.7, E11.2-E11.5, E11.7, E12.2-E12.5, E12.7, E13.2-E13.5, E13.7, E14.2-E14.5, E14.7	250.4-250.7
Hemiplegia or paraplegia	344.1, 342.x	G04.1, G11.4, G80.1, G80.2, G81.x, G82.x, G83.0-G83.4, G83.9	334.1, 342.x, 343.x, 344.0-344.6, 344.9
Renal disease	582.x, 583-583.7, 585.x, 586.x, 588.x	I12.0, I13.1, N03.2-N03.7, N05.2-N05.7, N18.x, N19.x, N25.0, Z49.0-Z49.2, Z94.0, Z99.2	403.01, 403.11, 403.91, 404.02, 404.03, 404.12, 404.13, 404.92, 404.93, 582.x, 583.0-583.7, 585.x, 586.x, 588.0, V42.0, V45.1, V56.x
Any malignancy, including lymphoma and leukemia, except malignant neoplasm of skin	140.x-172.x, 174.x-195.8, 200.x-208.x	C00.x-C26.x, C30.x-C34.x, C37.x-C41.x, C43.x, C45.x-C58.x, C60.x-C76.x, C81.x-C85.x, C88.x, C90.x-C97.x	140.x-172.x, 174.x-195.8, 200.x-208.x, 238.6
Moderate or severe liver disease	456.0-456.21, 572.2-572.8	I85.0, I85.9, I86.4, I98.2, K70.4, K71.1, K72.1, K72.9, K76.5, K76.6, K76.7	456.0-456.2, 572.2-572.8
Metastatic solid tumor	196.x-199.1	C77.x-C80.x	196.x-199.x
AIDS/HIV	042.x-044.x	B20.x-B22.x, B24.x	042.x-044.x

Source:

\+ Deyo RA, Cherkin DC, Ciol MA. Adapting a clinical comorbidity index for use with ICD-9-CM administrative databases. *J Clin Epidemiol.* 1992; 45: 613-9.

* Quan H, Sundararajan V, Halfon P, et al. Coding algorithms for defining Comorbidities in ICD-9-CM and ICD-10 administrative data. *Med Care.* 2005 Nov; 43(11): 1130-9.

TABLE 1. ICD-9-CM and ICD-10 Coding Algorithms for Charlson Comorbidities

Comorbidities	Deyo's ICD-9-CM +	ICD-10 *	Enhanced ICD-9-CM *
Myocardial infarction	410.x, 412.x	I21.x, I22.x, I25.2	410.x, 412.x
Congestive heart failure	428.x	I09.9,I11.0, I13.0, I13.2, I25.5, I42.0, I42.5-I42.9, I43.x, I50.x, P29.0	398.91, 402.01, 402.11, 402.91, 404.01, 404.03, 404.11, 404.13, 404.91, 404.93, 425.4-425.9, 428.x
Peripheral vascular disease	443.9, 441.x, 785.4, V43.4 Procedure 38.48	I70.x, I71.x, I73.1, I73.8, I73.9, I77.1, I79.0, I79.2, K55.1, K55.8, K55.9, Z95.8, Z95.9	093.0, 437.3, 440.x, 441.x, 443.1-443.9, 447.1, 557.1, 557.9, V43.4
Cerebrovascular disease	430.x-438.x	G45.x, G46.x, H34.0, I60.x-I69.x	362.34, 430.x-438.x
Dementia	290.x	F00.x-F03.x, F05.1, G30.x, G31.1	290.x, 294.1, 331.2
Chronic pulmonary disease	490.x-505.x, 506.4	I27.8, I27.9, J40.x-J47.x, J60.x-J67.x, J68.4, J70.1, J70.3	416.8, 416.9, 490.x-505.x, 506.4, 508.1, 508.8
Rheumatic disease	710.0, 710.1, 710.4, 714.0-714.2, 714.81, 725.x	M05.x, M06.x, M31.5, M32.x-M34.x, M35.1, M35.3, M36.0	446.5, 710.0-710.4, 714.0-714.2, 714.8, 725.x
Peptic ulcer disease	531.x-534.x	K25.x-K28.x	531.x-534.x
Mild liver disease	571.2, 571.4-571.6	B18.x, K70.0-K70.3, K70.9, K71.3-K71.5, K71.7, K73.x, K74.x, K76.0, K76.2-K76.4, K76.8, K76.9, Z94.4	070.22, 070.23, 070.32, 070.33, 070.44, 070.54, 070.6, 070.9, 570.x, 571.x, 573.3, 573.4, 573.8, 573.9, V42.7
Diabetes without chronic complication	250.0-250.3, 250.7	E10.0, E10.l, E10.6, E10.8, E10.9, E11.0, E11.1, E11.6, E11.8, E11.9, E12.0, E12.1, E12.6, E12.8, E12.9, E13.0, E13.1, E13.6, E13.8, E13.9, E14.0, E14.1, E14.6, E14.8, E14.9	250.0-250.3, 250.8, 250.9
Diabetes with chronic complication	250.4-250.6	E10.2-E10.5, E10.7, E11.2-E11.5, E11.7, E12.2-E12.5, E12.7, E13.2-E13.5, E13.7, E14.2-E14.5, E14.7	250.4-250.7
Hemiplegia or paraplegia	344.1, 342.x	G04.1, G11.4, G80.1, G80.2, G81.x, G82.x, G83.0-G83.4, G83.9	334.1, 342.x, 343.x, 344.0-344.6, 344.9
Renal disease	582.x, 583-583.7, 585.x, 586.x, 588.x	I12.0, I13.1, N03.2-N03.7, N05.2-N05.7, N18.x, N19.x, N25.0, Z49.0-Z49.2, Z94.0, Z99.2	403.01, 403.11, 403.91, 404.02, 404.03, 404.12, 404.13, 404.92, 404.93, 582.x, 583.0-583.7, 585.x, 586.x, 588.0, V42.0, V45.1, V56.x
Any malignancy, including lymphoma and leukemia, except malignant neoplasm of skin	140.x-172.x, 174.x-195.8, 200.x-208.x	C00.x-C26.x, C30.x-C34.x, C37.x-C41.x, C43.x, C45.x-C58.x, C60.x-C76.x, C81.x-C85.x, C88.x, C90.x-C97.x	140.x-172.x, 174.x-195.8, 200.x-208.x, 238.6
Moderate or severe liver disease	456.0-456.21, 572.2-572.8	I85.0, I85.9, I86.4, I98.2, K70.4, K71.1, K72.1, K72.9, K76.5, K76.6, K76.7	456.0-456.2, 572.2-572.8
Metastatic solid tumor	196.x-199.1	C77.x-C80.x	196.x-199.x
AIDS/HIV	042.x-044.x	B20.x-B22.x, B24.x	042.x-044.x

Source:

\+ Deyo RA, Cherkin DC, Ciol MA. Adapting a clinical comorbidity index for use with ICD-9-CM administrative databases. *J Clin Epidemiol.* 1992; 45: 613-9.

* Quan H, Sundararajan V, Halfon P, et al. Coding algorithms for defining Comorbidities in ICD-9-CM and ICD-10 administrative data. *Med Care.* 2005 Nov; 43(11): 1130-9.
